# Supplementary material for: In or Out? New Insights on Exon Recognition through Splice-Site Interdependency
Source: Int J Mol Sci. 2020 Mar 26;21(7):2300. doi: 10.3390/ijms21072300 (PMC7177576; doi:10.3390/ijms21072300)
Supplement: Supplementary file 1 [file ijms-21-02300-s001.zip › 03_Supplemental data/Supplementary Fig S1.pptx]

## Slide 1
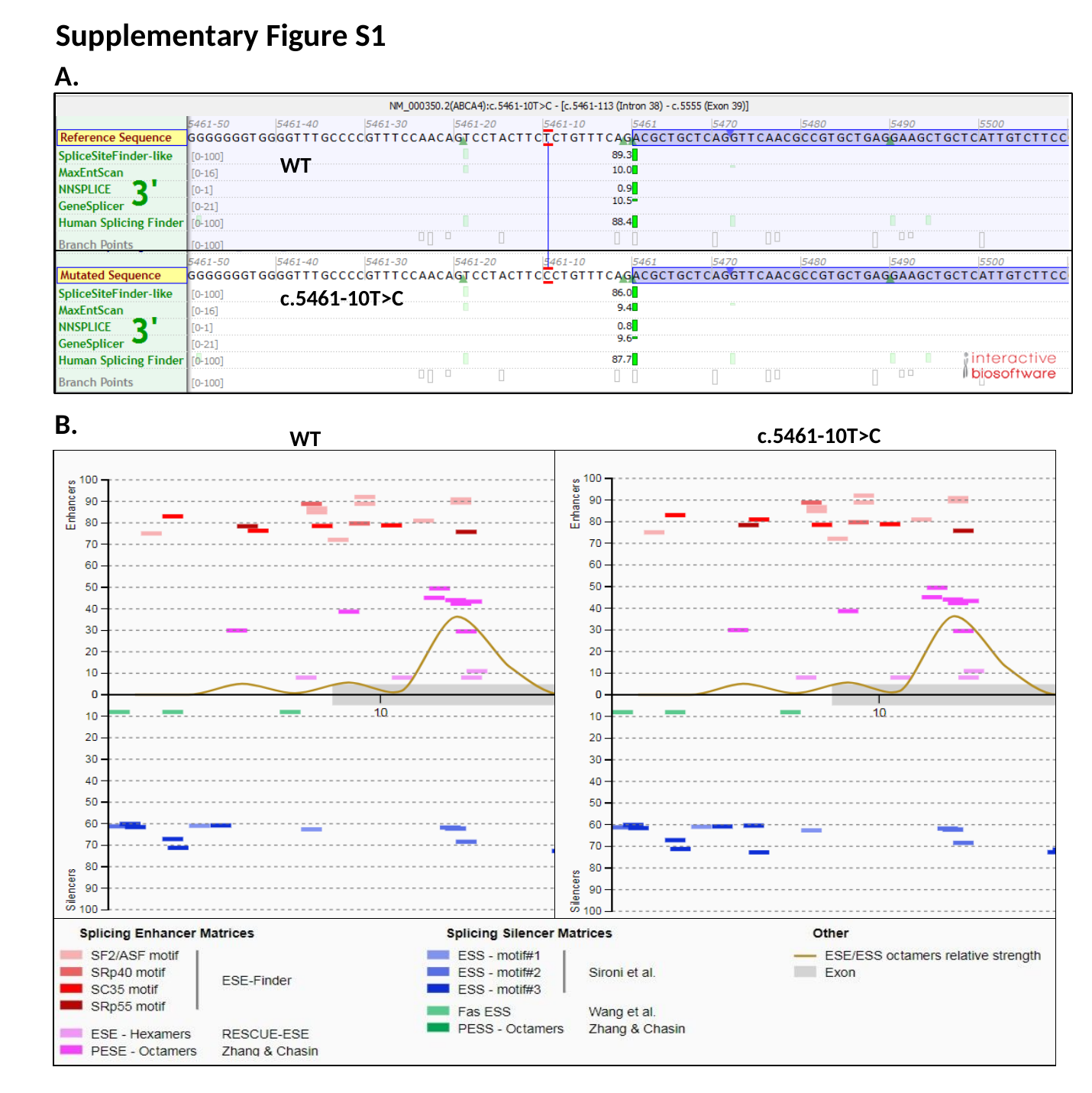

Supplementary Figure S1
A.
WT
c.5461-10T>C
B.
c.5461-10T>C
WT
| | |
| --- | --- |
| | |

## Slide 2
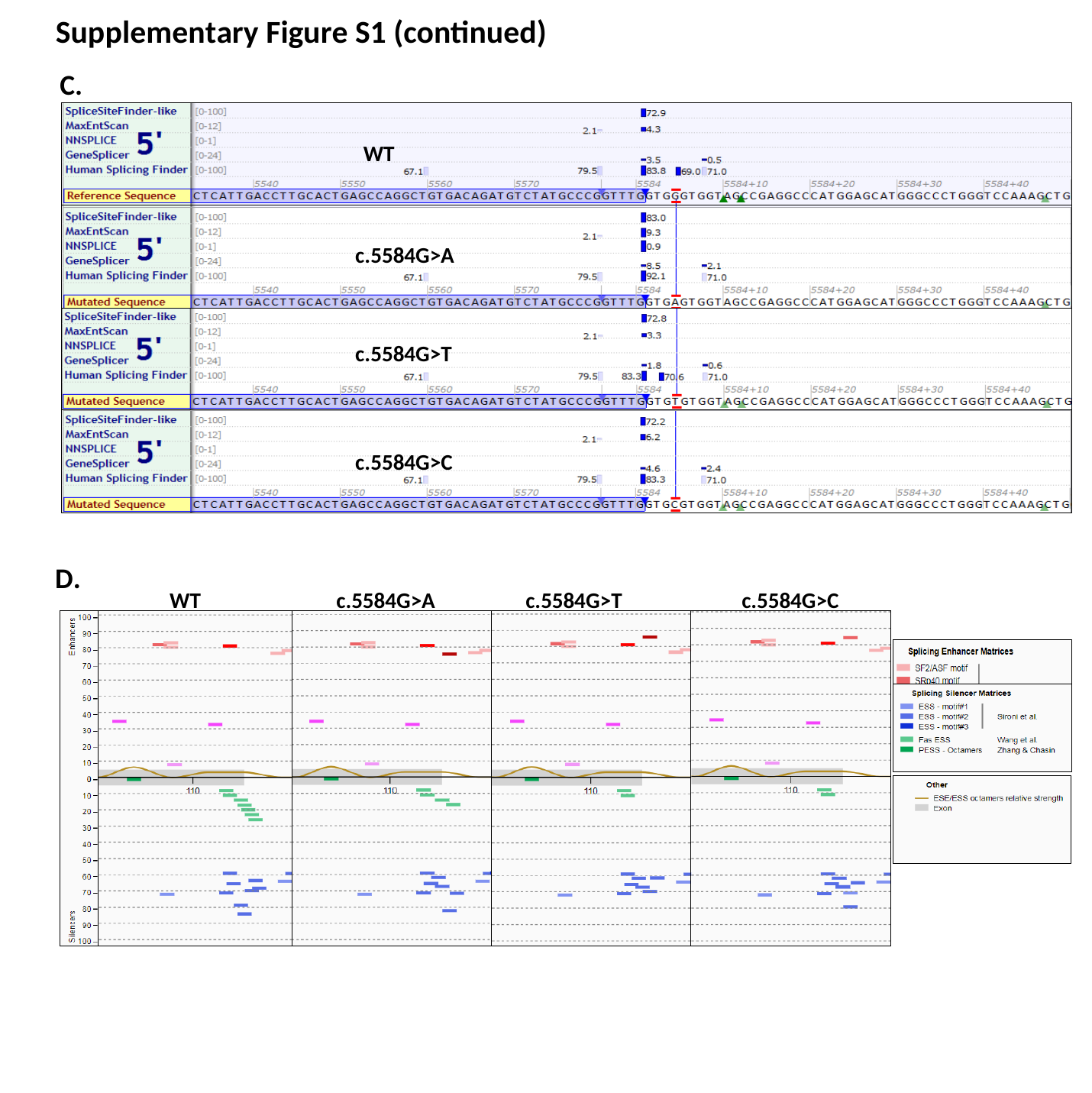

Supplementary Figure S1 (continued)
C.
WT
c.5584G>A
c.5584G>T
c.5584G>C
c.5584G>A
c.5584G>T
c.5584G>C
D.
WT
| | | | | |
| --- | --- | --- | --- | --- |
