# Supplementary material for: In or Out? New Insights on Exon Recognition through Splice-Site Interdependency
Source: Int J Mol Sci. 2020 Mar 26;21(7):2300. doi: 10.3390/ijms21072300 (PMC7177576; doi:10.3390/ijms21072300)
Supplement: Supplementary file 1 [file ijms-21-02300-s001.zip › 03_Supplemental data/Supplementary Fig S2.pptx]

## Slide 1
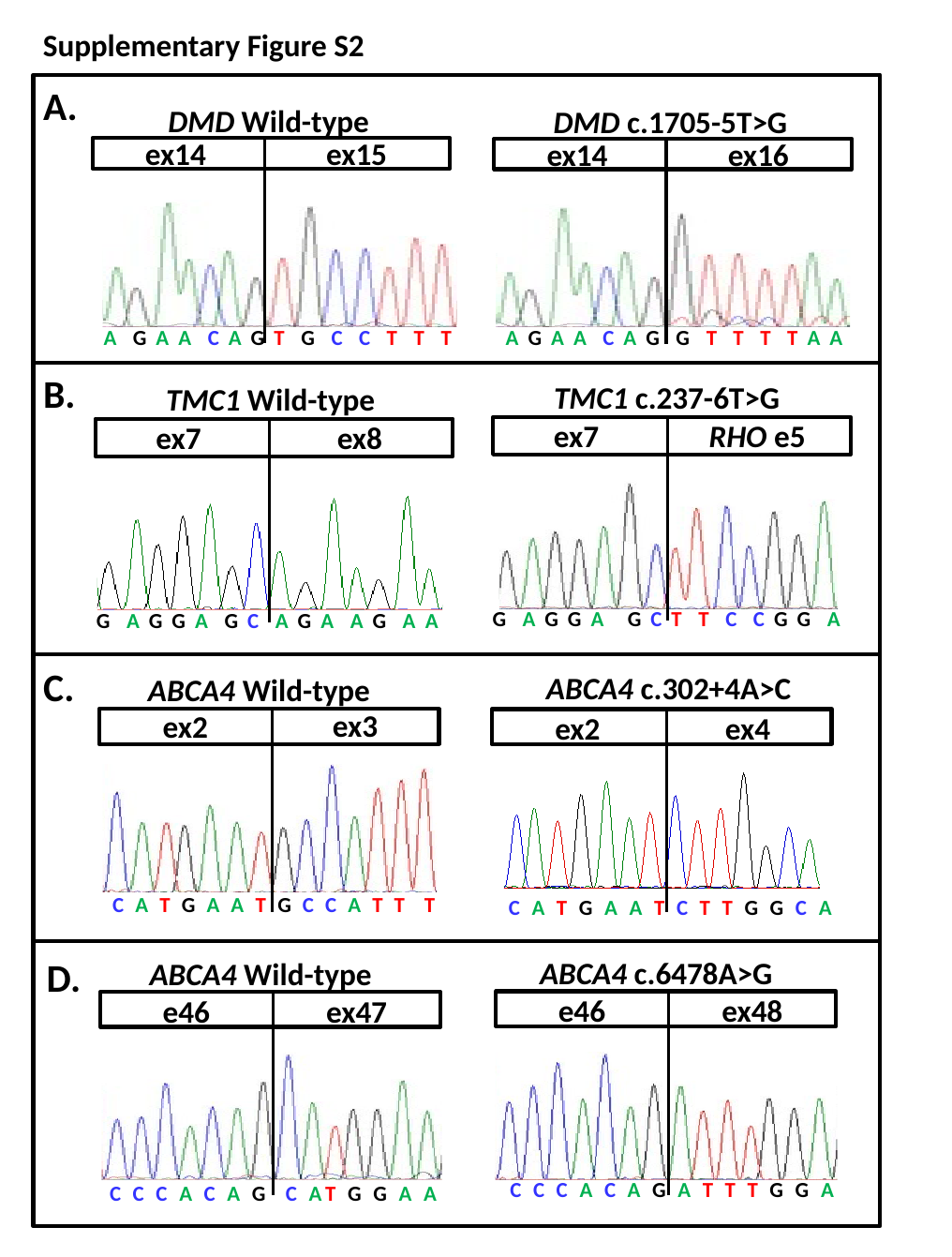

Supplementary Figure S2
A.
DMD Wild-type
ex14
ex15
 A G A A C A G T G C C T T T
DMD c.1705-5T>G
ex14
ex16
 A G A A C A G G T T T T A A
B.
TMC1 c.237-6T>G
ex7
RHO e5
G A G G A G C T T C C G G A
TMC1 Wild-type
ex7
ex8
G A G G A G C A G A A G A A
C.
ABCA4 c.302+4A>C
ex4
ex2
 C A T G A A T C T T G G C A
ABCA4 Wild-type
ex3
ex2
C A T G A A T G C C A T T T
D.
ABCA4 c.6478A>G
ex48
e46
 C C C A C A G A T T T G G A
ABCA4 Wild-type
ex47
e46
 C C C A C A G C AT G G A A
