# Supplementary material for: In or Out? New Insights on Exon Recognition through Splice-Site Interdependency
Source: Int J Mol Sci. 2020 Mar 26;21(7):2300. doi: 10.3390/ijms21072300 (PMC7177576; doi:10.3390/ijms21072300)
Supplement: Supplementary file 1 [file ijms-21-02300-s001.zip › 03_Supplemental data/Supplementary figure legends_13-03-2020_FC.docx]

**Supplementary figure legends**

**Figure S1. Splicing predictions of *ABCA4* exon 39 variant c.5461-10T>C and rescue variants.** **A**. Splicing predictions from Alamut Visual algorithms for the wild-type (WT) sequence and c.5461-10T>C. **B**. Exonic splicing enhancer (ESE) and exonic splicing silencer (ESS) motifs in exon 39 SAS according to Human Splicing Finder for the WT and mutant sequences. Similarly, all five Alamut splice-site strength algorithms and Human Splicing Finder ESE and ESS predictions are shown for the rescue variants, c.5584+4G>T, c.5584+4G>C and c.5584+4G>A, in panels **C.** and **D.**, respectively.

**Figure S2: Sanger sequencing results of cDNA from *in vitro* splice assays carrying segments of *ABCA4*, *DMD*, and *TMC1*.** **A.** Normal *DMD* exon 14-exon 15 splicing when using wild-type (WT) construct and exon 15 skipping due to variant c.1705-5T>G. **B.** Normal *TMC1* exon 7-exon 8 splicing for WT construct and exon 8 skipping due to c.237-6T>G. **C.** Normal *ABCA4* exon 2-exon 3 splicing for WT construct and exon 3 skipping due to variant c.302+4A>C. **D.** Normal *ABCA4* exon 46-exon 47 splicing for WT construct and exon 47 skipping due to variant c.6478A>G.

**Figure S3. Splicing predictions of *DMD* exon 15 variant c.1705-5T>G** **and a rescue variant**. **A**. Splicing predictions from Alamut Visual algorithms for the wild-type (WT) sequence and c.1705-5T>G. **B**. Exonic splicing enhancer (ESE) and exonic splicing silencer (ESS) motifs in exon 15 SAS according to Human Splicing Finder for the WT and mutant sequences. Similarly, all five Alamut splice-site strength algorithms and Human Splicing Finder ESE and ESS predictions are shown for the rescue variant c.1812+4T>A, in panels **C.** and **D.**, respectively.

**Figure S4.** **Splicing predictions of *TMC1* exon 8 variant c.237-6T>G and rescue variants**. **A**. Splicing predictions from Alamut Visual algorithms for the wild-type (WT) sequence and c.237-6T>G. **B**. Exonic splicing enhancer (ESE) and exonic splicing silencer (ESS) motifs in exon 8 SAS according to Human Splicing Finder for the WT and mutant sequence. Similarly, all five Alamut splice-site strength algorithms and Human Splicing Finder ESE and ESS predictions are shown for the rescue variant c.362+4T>A, in panels **C.** and **D.**, respectively.

**Figure S5.** **Splicing predictions of *ABCA4* exon 3 variant c.302+4A>C and a rescue variant**. **A**. Splicing predictions from Alamut Visual algorithms for the wild-type (WT) sequence and c.302+4A>C. **B**. Exonic splicing enhancer (ESE) and exonic splicing silencer (ESS) motifs in the 5’ splice site of exon 3 according to Human Splicing Finder for the WT and mutant sequence. Similarly, all five Alamut splice-site strength algorithms and Human Splicing Finder ESE and ESS predictions are shown for the rescue variant c.161-3A>C, in panels **C.** and **D.**, respectively.

**Figure S6.** **Splicing predictions of *ABCA4* exon 47 variant c.6478A>G and a rescue variant**. **A**. Splicing predictions from Alamut Visual algorithms for the wild-type (WT) sequence and c.6478A>G. **B**. Exonic splicing enhancer (ESE) and exonic splicing silencer (ESS) motifs in the 5’ splice site of exon 47 according to Human Splicing Finder for the WT and mutant sequence. Similarly, all five Alamut splice-site strength algorithms and Human Splicing Finder predictions are shown for the rescue variant c.6387C>G, in panels **C.** and **D.**, respectively.

**References:**

1. Desmet, F.-O.; Hamroun, D.; Lalande, M.; Collod-Béroud, G.; Claustres, M.; Béroud, C., Human Splicing Finder: an online bioinformatics tool to predict splicing signals. *Nucleic acids research* **2009,** 37, (9), e67-e67.
