# Supplementary figures and images for: In or Out? New Insights on Exon Recognition through Splice-Site Interdependency
Source: Int J Mol Sci. 2020 Mar 26;21(7):2300. doi: 10.3390/ijms21072300 (PMC7177576; doi:10.3390/ijms21072300)

## Slide 1
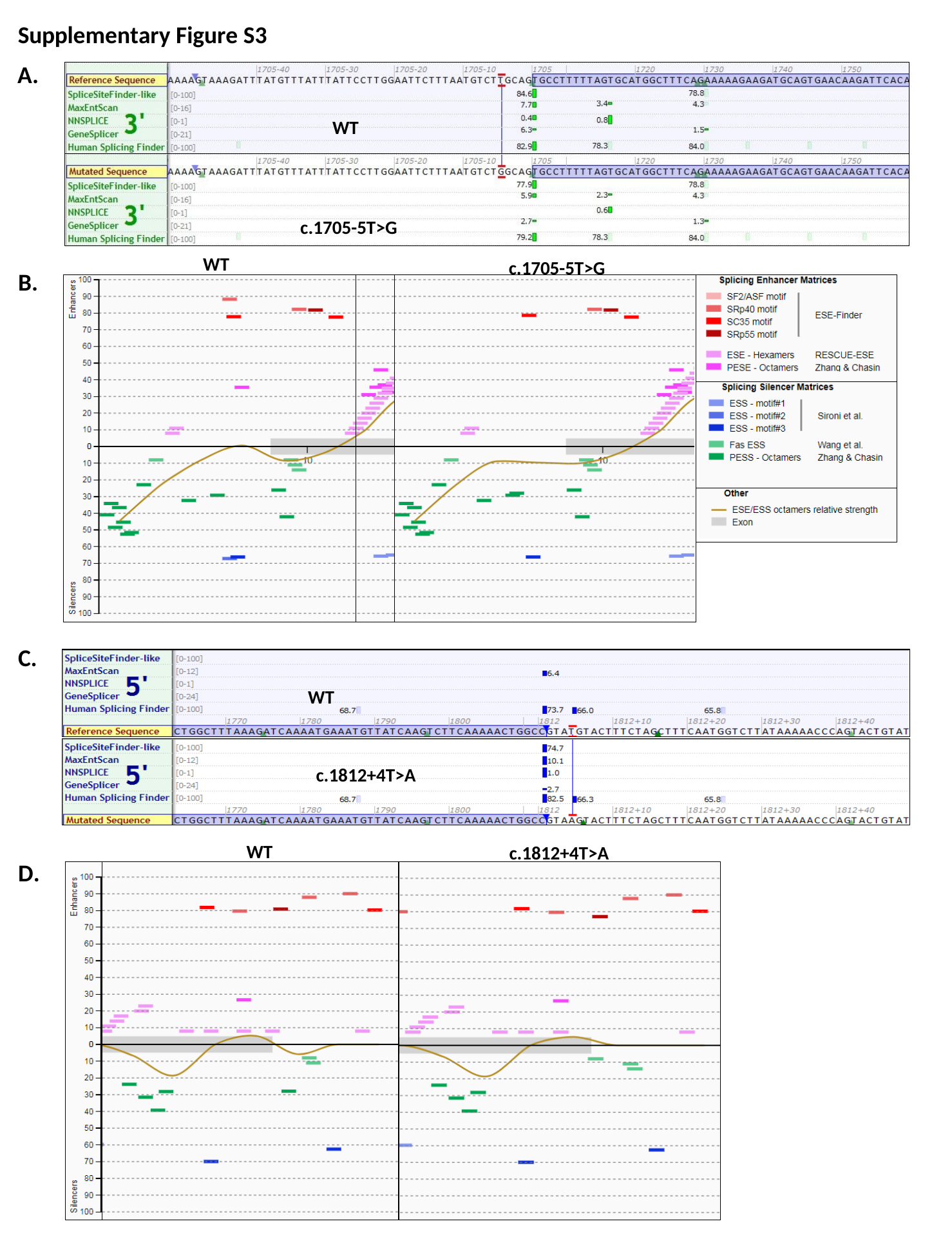

Supplementary Figure S3
A.
WT
WT
c.1705-5T>G
c.1705-5T>G
B.
| | | | |
| --- | --- | --- | --- |
| | | | |
| | | | |
| | | | |
C.
WT
c.1812+4T>A
WT
c.1812+4T>A
D.

Supplement: Supplementary file 1 [file ijms-21-02300-s001.zip › 03_Supplemental data/Supplementary Fig S3.pptx]

## Slide 1
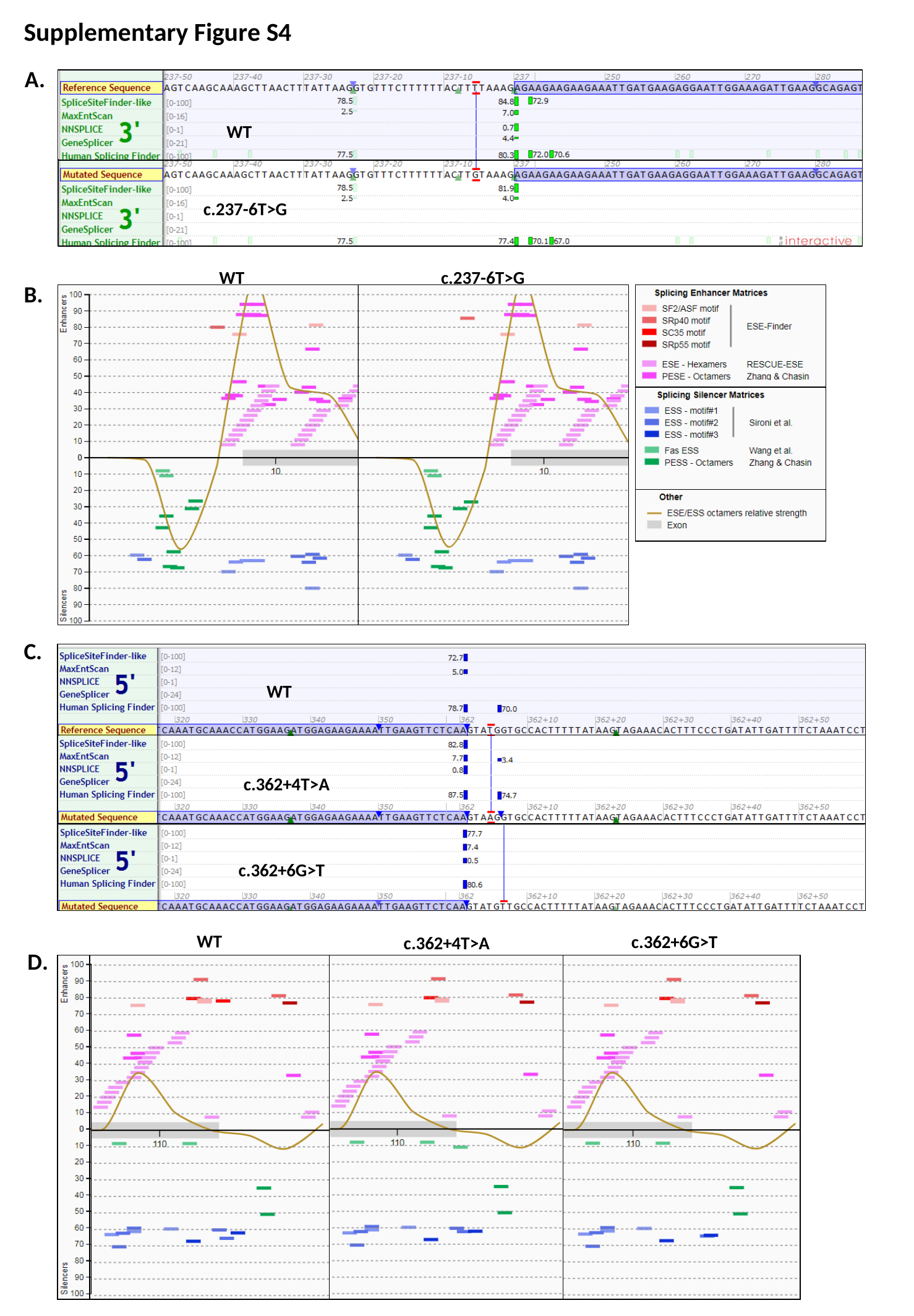

Supplementary Figure S4
A.
WT
c.237-6T>G
WT
c.237-6T>G
B.
C.
| |
| --- |
| |
| |
WT
c.362+4T>A
c.362+6G>T
c.362+6G>T
c.362+4T>A
WT
D.
| | | | |
| --- | --- | --- | --- |

Supplement: Supplementary file 1 [file ijms-21-02300-s001.zip › 03_Supplemental data/Supplementary Fig S4.pptx]

## Slide 1
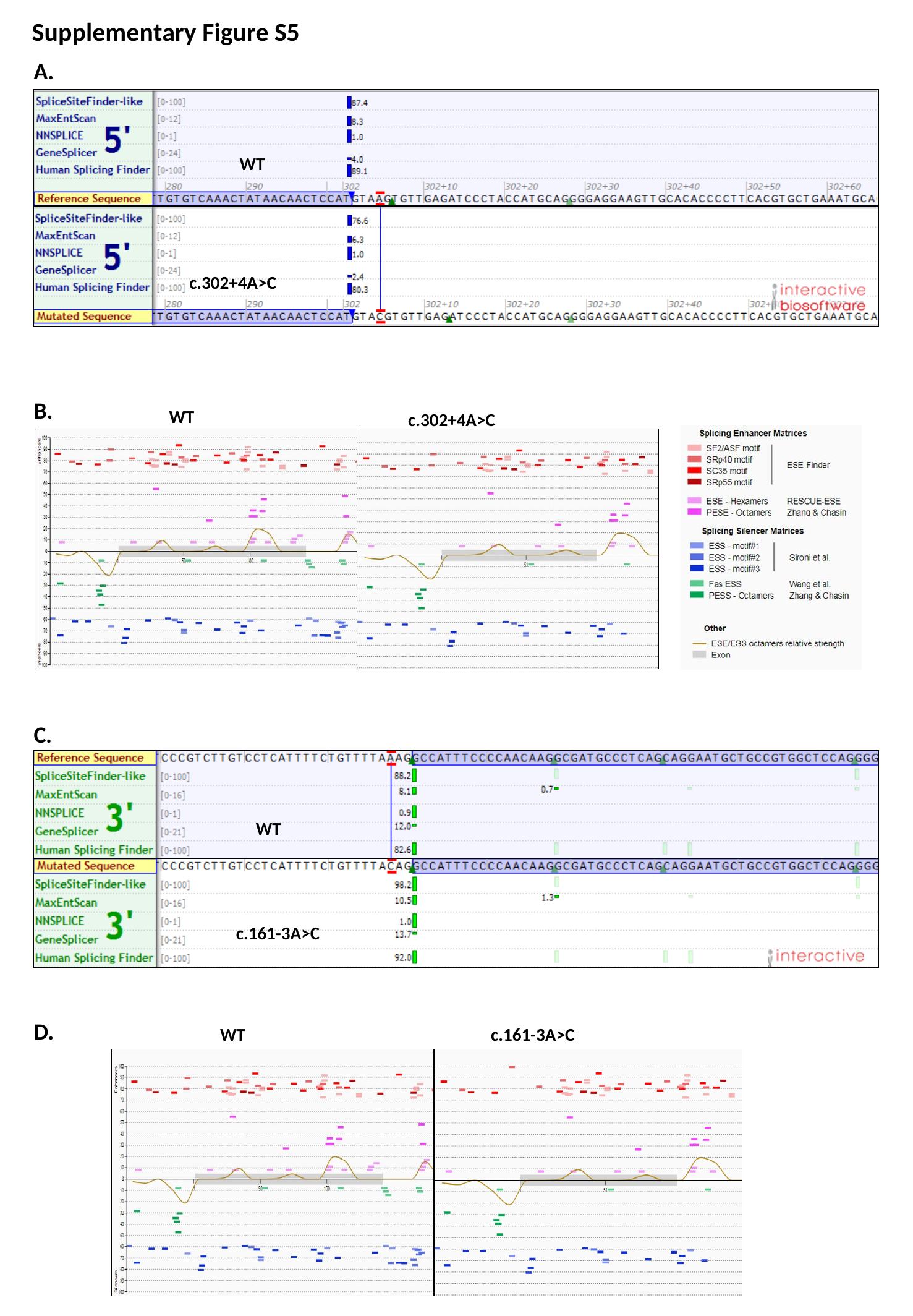

Supplementary Figure S5
A.
WT
c.302+4A>C
B.
WT
c.302+4A>C
C.
WT
c.161-3A>C
D.
WT
c.161-3A>C

Supplement: Supplementary file 1 [file ijms-21-02300-s001.zip › 03_Supplemental data/Supplementary Fig S5.pptx]

## Slide 1
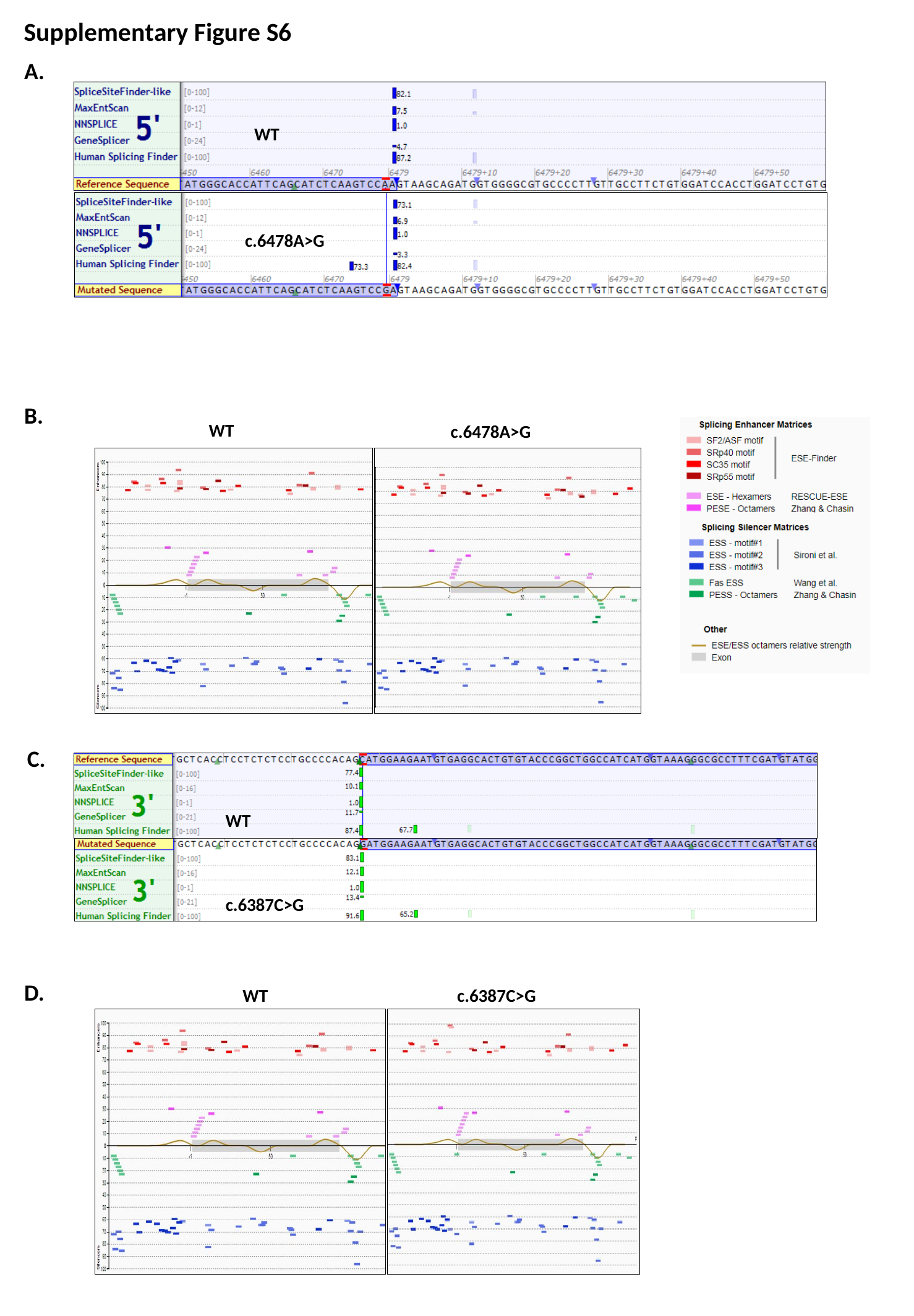

Supplementary Figure S6
A.
WT
c.6478A>G
B.
WT
c.6478A>G
C.
WT
c.6387C>G
D.
WT
c.6387C>G

Supplement: Supplementary file 1 [file ijms-21-02300-s001.zip › 03_Supplemental data/Supplementary Fig S6.pptx]
